# Biomimetic catechol-based adhesive polymers for dispersion of polytetrafluoroethylene (PTFE) nanoparticles in an aqueous medium†

**DOI:** 10.1039/c9ra10606e

**Published:** 2020-01-23

**Authors:** Manjit Singh Grewal, Hiroshi Yabu

**Affiliations:** WPI-Advanced Institute of Materials Research (WPI-AIMR), Tohoku University 2-1-1, Katahira Aoba-Ku Sendai 980-8577 Japan hiroshi.yabu.d5@tohoku.ac.jp

## Abstract

Biomimetic synthetic functional materials are valuable for a large number of practical applications with improved or tunable performance. In this paper, we present a series of mussel-inspired biomimetic catechol-containing copolymers synthesized from dopamine methacrylamide (DMA) and 2-(2-ethoxyethoxy)ethyl acrylate (EEA) and abbreviated as poly(PDMA-PEEA). The successfully synthesized adhesive polymers allow adhering polytetrafluoroethylene (PTFE) and were used for coating PTFE particles in organic solvent and re-dispersion in an aqueous medium. Adhesive polymer coated PTFE particles were efficiently used as a nanoreactor for generating silver (Ag) metal nanoparticles (NPs).

## Abstract 2

Biomimetic synthetic functional materials are valuable for a large number of practical applications with improved or tunable performance.

## Graphical abstract

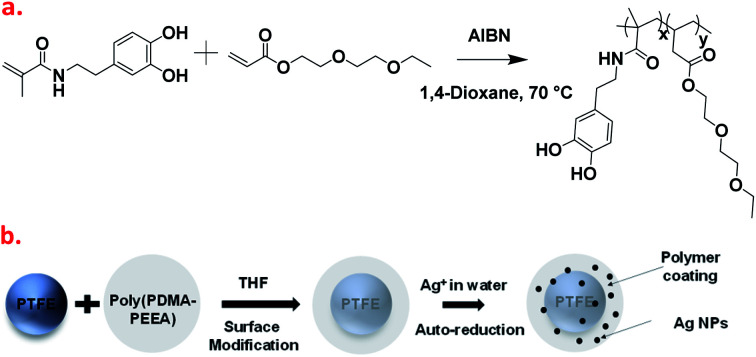

## Introduction

Mussels derived adhesive proteins which contain catecholic amino acids, 3,4-dihydroxy-l-phenylalanine (DOPA), are useful materials for industrial, biomedical and pharmaceutical applications owing to their characteristic binding applicability, hydrophilicity, permeability, and stability towards a wide variety of surfaces both in air and water.^1–3^ Catechol moieties in DOPA has a well-known ability to form various types of chemical interactions, *e.g.*, cross-linking by oxidation, and hence, shows excellent adhesion properties for a wide variety of materials, such as inorganic metals, metal oxides, alloys, polymers, glass, wood and ceramics.^4,5^ Strong research efforts have been made in the last decade to develop new biomimetic catechol based adhesive materials for better adhesion of components and surface coatings or modifiers motivated by the growing needs and demands of the automotive and aerospace industries.^6,7^ Polymers containing catechol groups have been reportedly used for surface modification,^8–10^ surface coatings,^11–13^ stabilization of nanoparticles,^14–16^ and adhesives.^17,18^

PTFE, which is a synthetic hydrophobic fluoropolymer of tetrafluoroethylene, has several successful engineering practical applications such as non-stick coatings for cookware and other appliances, as friction-reducing lubricants, graft materials in biomedical field, chemical and electronic fields,^19–21^ due to its attractive properties of low surface energy, high thermal and chemical stability, low dielectric constant, low water adsorption and high potential biological inertness. However, high hydrophobicity and poor wettability of PTFE limits its performance in real applications. Various reinforcement and modification of PTFE such as coating with functional polymer,^25,26^ chemical etching,^27^ ion-beam,^28^ ozone treatment,^29^ plasma modifications^30^ have been tried to intrinsically improve the material performance. Many of the above-mentioned approaches require complicated processes and specially made instruments. To date, there has been a large number of polymer materials which could disperse extremely hydrophobic and poorly adhesive PTFE^22–24^ in organic solvents, however, PTFE dispersion in an aqueous medium without using fluorinated surfactants or additional fluorinated reagents remains a challenging scope. Inspired from the reducing properties of catechol moieties, the composite materials prepared by coating PTFE particles in nm range coated with amphiphilic polymers containing catechol moieties can be exploited as nanosized reactors that can automatically reduce metal ions.

**Fig. 1:**
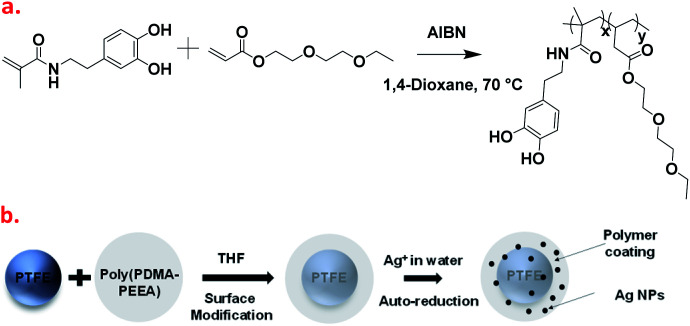
(a) Reaction scheme for the synthesis of co-polymer, poly(PDMA–PEEA). (b) Sketched representation of coating of the PTFE nanoparticles with the adhesive polymer in THF solvent (surface modification), the addition of Ag^+^ ions in the aqueous medium containing polymer-coated PTFE particles, and plausible illustration of reduced Ag nanoparticles anchored on to hydrophilic catechol surface.

In the present study, a series of adhesive copolymers, poly(PDMA–PEEA) based on dopamine methacrylamide (DMA) and 2-(2-ethoxyethoxy)ethyl acrylate (EEA) was synthesized and modified by varying copolymerization ratios. The synthesized polymers, abbreviated as poly(PDMA–PEEA) show strong adhesion properties on PTFE substrates; and the surfaces of hydrophobic PTFE particles were successfully modified with those copolymers in tetrahydrofuran solution. The surface-modified PTFE particles can be dispersed in many kinds of solvents, especially in aqueous media. Furthermore, the remaining catechol groups on the surface of modified PTFE particles show reduction property of silver ions and formed silver nanoparticle shells on the surface of them (Fig. 1). Results of silver ions reduction allow tailoring the design of such composite materials as nanoreactors to replace, totally or partially expensive catalytic materials to ensure electrochemical operation. We envisage that this new class of composite materials with simple and easy preparation will facilitate tuning of properties for a host of other polymer-nanoparticles as nanoreactors without any additional reducing agents.

## Experimental

### Materials

Dopamine methacrylamide (DMA, TCI, Tokyo, Japan), 2-(2-ethoxyethoxy)ethyl acrylate (EEA, >98% purity, TCI, Tokyo, Japan), 2,2′-azobis(2-methylpropionitrile) (AIBN, 98% purity, Sigma Aldrich, St. Louis, MO, USA), super-dehydrated 1,4-dioxane (Fujifilm Wako Pure Chemical Co.) were used as received. Polytetrafluoroethylene (PTFE) nanoparticles with average diameter of 100 nm were purchased from Okuno Chemicals Industries Co., Ltd., Osaka, Japan.

### Methods

^1^H NMR spectra of a series of adhesive polymers were performed by a Bruker ^1^H NMR spectroscopy at 400 MHz (AVANCE III spectrometer, Bruker, Germany) using tetramethylsilane (*δ* = 0.00 ppm) as an internal standard. Deuterated dimethylsulfoxide (DMSO-d_6_) was used as the solvent. Molecular weights of the adhesive polymers were determined using GPC performed on a Tosoh HLC-8320GPC, with THF as an eluent and polystyrene as a standard. The FT-IR spectra were recorded with a JASCO FT/IR-6100 between 4000 and 600 cm^−1^ using a diamond-attenuated total reflection (ATR) accessory. UV-Vis spectrum was measured by JASCO V-670 spectrophotometer at a room temperature over the range 200–800 nm using the polymer solution in THF (20 mg mL^−1^). Thermogravimetry/differential thermal analysis (TG-DTAA) was performed on RIGAKU Thermo plus EvoII TG-DTA8210, whereas differential scanning calorimetry (DSC) was performed on Mettler Toledo, Japan at a heating rate of 10 °C min^−1^ under a nitrogen atmosphere. The polymer specimens were dried at 60 °C for overnight under vacuum to remove any absorbed moisture before TGA and DSC. The force–displacement measurements were carried out by coating adhesive polymer on to rectangular glass or polytetrafluoroethylene substrates with 3 cm × 1 cm dimensions pulled apart at an elongation speed of 1.0 mm min^−1^ on a IMADA test stand at room temperature. Morphology of PTFE and coated nanoparticles were observed by using scanning electron microscopy, SEM (JEOL JSM-6700F). The transmission electron microscopy (TEM) analysis were operated on a JEOL JSM-2100F system to observe the state of dispersion of PTFE nanoparticles by coating with the adhesive poly(PDMA–PEEA) polymer. DLS employing a Zetasizer Nano ZS (Malvern ZEN3600) to observe the particle size distribution.

### Preparation of adhesive co-polymer, poly(PDMA–PEEA)

Fig. 1a shows the synthetic route of copolymerization between DMA and EEA *via* thermally initiated free radical polymerization from a homogeneous precursor solution composed of DMA, EEA, and thermal initiator (AIBN) according to the previous studies.^31,32^ The synthesized co-polymer was abbreviated as poly(PDMA–PEEA). In order to investigate any change in thermo-mechanical properties by enhancing the mol% of DMA, a series of copolymers was prepared wherein the weight ratio of DMA was kept constant. The weight ratio of DMA and EEA in copolymers varied from 1 : 2.5, 1 : 5.0, 1 : 7.2 and 1 : 10. A brief polymerization procedure for poly(PDMA–PEEA) with DMA : EEA as 1 : 7.2 is described as follows: DMA (0.20 g, 0.9 mmol), EEA (1.43 g, 7.61 mmol) and AIBN (15 mg) in 40 mL of dehydrated 1,4-dioxane as solvent were mixed in a 2-neck round bottom flask under inert nitrogen atmosphere and refluxed at 70 °C for 12–15 hours. The reaction mixture was diluted with 20 mL of 1,4-dioxane and then added to 500 mL of hexane to precipitate the polymers. A colorless viscous gel was obtained which was dried in vacuum oven at 60 °C overnight and kept in desiccator for further characterizations. Analogous procedure was used to prepare 1 : 2.5, 1 : 5, and 1 : 10 variations of poly(PDMA–PEEA).

### Preparation of water-dispersive polytetrafluoroethylene particles in the presence of poly(PDMA–PEEA)

Direct dispersion of PTFE particles in aqueous medium is difficult even with prolonged sonication. In our method, we used an indirect approach where PTFE particles are first uniformly coated with adhesive polymer in an organic solvent. In a typical procedure, 3 mL of THF solution containing poly(PDMA–PEEA) (10 mg mL^−1^) was poured onto a 3 mL of THF solution containing PTFE particles (5 mg mL^−1^). The mixture was stirred using homogenizer for 5 minutes. The polymer coated PTFE particles were centrifugally separated at 10 000 rpm and washed three times with water by applying repeated centrifugation. The product particles were then suspended in 5 mL of water and ultrasonicated for 2 minutes to separate large aggregates if formed. The clear solution containing suspended PTFE particles coated with adhesive polymer, namely PTFE@poly(PDMA–PEEA) was obtained which was further used for particle characterization. Fig. S1† shows the representative photograph of adhesive polymer, poly(PDMA–PEEA) 1 : 10 and poly(PDMA–PEEA) coated PTFE particles and PTFE particles in aqueous solution.

## Results and discussion

A series of adhesive polymer, poly(PDMA–PEEA) was synthesized using DMA and EEA as monomers using thermal initiated free-radical polymerization as shown in Fig. 1a. The chemical structure of all the polymers were determined using ^1^H-NMR (Fig. S2†). The location of proton peaks in all adhesive polymers were similar, which indicated that they all had the similar chemical shifts in the main structure. Peaks were attributed at around 8.64, 6.40–6.63 ppm, 4.10 ppm, 3.37–3.57 ppm, 2.27–2.51 ppm, 1.09–1.58 ppm, 0.86–0.88 ppm as per the chemical structure (Fig. 1a). The characteristics peaks of DOPA in the range of 6.40–6.63 ppm shows that DOPA groups were successfully grafted onto adhesive copolymers. Some minimal peaks above 8.64 ppm shows oxidized DOPA forms (quinone or semi-quinone).

The copolymerization was further investigated by FT-IR measurements. As shown in Fig. S3,† the decay of C=C stretching vibrations of methacrylate or acrylate group at 1638 cm^−1^ after polymerization, and the appearance of broad absorbance between 3600 and 3100 cm^−1^ which is ascribed to N–H/O–H stretching vibrations in catechol moiety in copolymer.

The weight-average molecular weight of (*M*_w_), the number-average molecular weight (*M*_n_) and the molecular weight distribution (PDI) were determined by GPC measurements and summarized in Table 1. Poly(PDMA–PEEA) had high *M*_w_ and *M*_n_ values in the range of 1.41 × 10^5^ to 1.97 × 10^5^ and 6.83 × 10^3^ to 4.61 × 10^5^, respectively, and the PDI ranged from 2.07 to 3.27. Corresponding unimodal plots from the GPC results are shown in Fig. S4.†

**Table 1:** Molecular weight of adhesive copolymers, poly(PDMA–PEEA) with different DMA and EEA ratios

Sample	*M*_n_ [kg mol^−1^]	*M*_w_ [kg mol^−1^]	*M*_w_/*M*_n_
1 : 2.5	6.82	14.1	2.1
1 : 5	13.6	33.6	2.5
1 : 7.2	79.2	19.7	2.5
1 : 10	18.7	61.2	3.3

The solubility of adhesive polymers were tested by dissolving them in three groups of solvents:^1^ nonpolar solvent such as: toluene, hexane, diethyl ether, dichloromethane,^2^ polar protic solvents such as distilled water, methanol, ethanol, isopropanol, and^3^ polar aprotic solvent such as tetrahydrofuran (THF), dimethyl formamide, dimethyl acetamide (DMAc), *N*-methyl-2-pyrrolidone, dimethyl sulfoxide, and dioxane at room temperature. All the adhesive polymers were soluble in polar aprotic solvent and insoluble in non-polar solvent. As the content of EEA in the copolymer increased, the adhesive polymer showed fair solubility in aqueous medium due to increase in hydrophilicity.

**Fig. 2:**
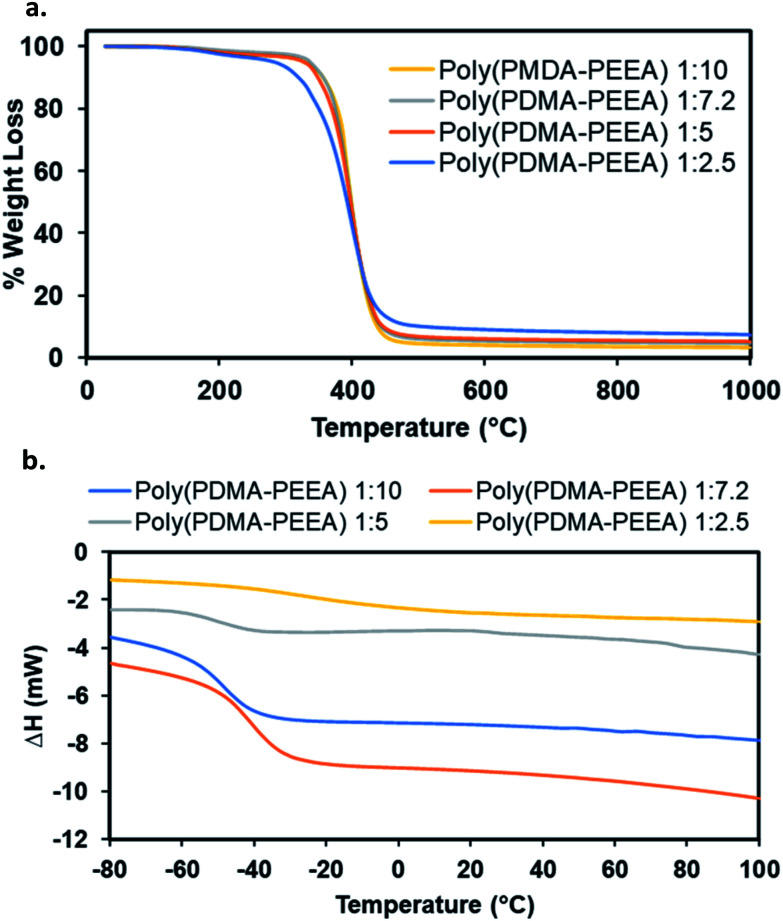
Thermal properties of adhesive copolymer, poly(PDMA–PEEA). (a) TGA and (b) DSC plots respectively.

The thermal degradation of all the adhesive polymers, poly(PDMA–PEEA) with different monomers ratio was investigated by thermogravimetric analysis (TGA) in a nitrogen atmosphere at a heating rate of 10 °C min^−1^, and the 5% and 10% weight-loss temperatures, *T*_5_ and *T*_10_, were determined (Fig. 2a). The slight weight loss of about 0.6% below 110 °C is due to the moisture that had been absorbed from the atmosphere during the handling of samples for testing. As revealed by the graphs, the main degradation of the adhesive copolymer from about 200–480 °C corresponds to the decomposition of DMA and EEA. Residual weight loss of 5% and 10% of the network polymers are in the ranges of 282.64–336.72 °C and 319.35–356.58 °C, respectively. Addition of higher amounts of EEA enhances the thermal stability of the co-polymer matrix possibly which is clearly visible in the thermograms. The overall weight loss is consistent with the copolymer composition taking into account the experimental errors related to the measurement and the sample preparation.

The differential scanning calorimetry (DSC) experiments were conducted to study the phase changes that were indicated by the glass transition temperature (*T*_g_) in the adhesive copolymers, poly(PDMA–PEEA). In a typical measurement, the polymer samples were heated at 10 °C min^−1^ from room temperature up to 100 °C as 1st run, then again cooled down to −100 °C as 2nd run and finally heated up to 150 °C as 3rd run. *T*_g_ values were calculated from the 3rd run as the cross-point of the heat capacity change observed during the transition from glassy state to rubbery state in the DSC trace. The DSC results are summarized in Table 2, and the corresponding profiles are shown in Fig. 2b. As evident in the DSC curves of all the polymer specimens, the *T*_g_ values ranged from −7.2 °C to −47.3 °C. The combined results of both DSC and TGA profiles show that no unusual phase changes or weight losses occur within the temperature range of 30 to 200 °C, which makes the adhesive copolymers thermally stable and useful over several synthetic adhesives which readily decomposes over lower range of temperatures.

**Table 2:** Thermal properties of poly(PDMA–PEEA) with different EEA contents as a measurements of force–displacement values

Sample	*T*_5_ [°C]	*T*_10_ [°C]	*T*_g_ [°C]
1 : 2.5	282	319	−7.2
1 : 5	327	348	−47.3
1 : 7.2	337	357	−39.6
1 : 10	328	356	−43.2

**Fig. 3:**
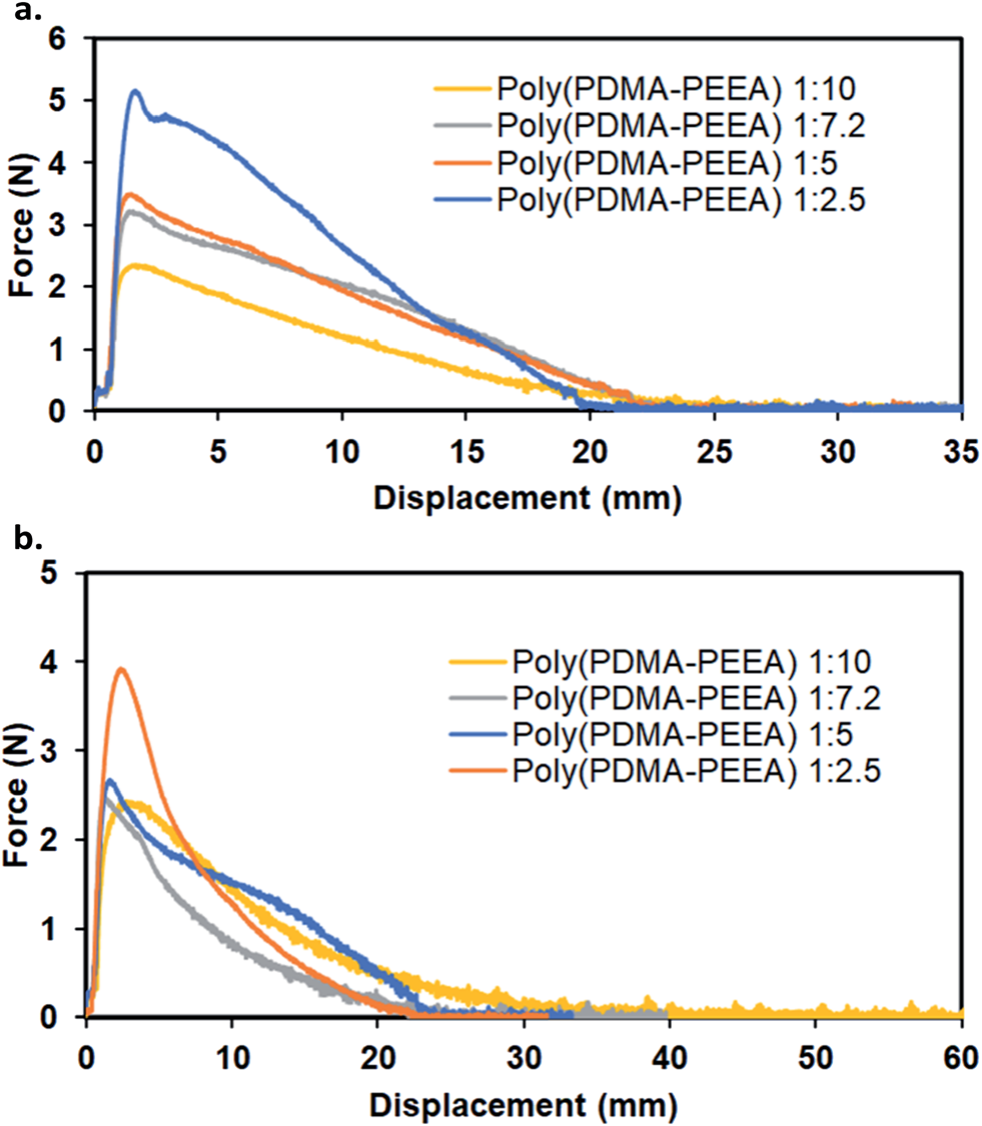
Mechanical properties of adhesive copolymer, poly(PDMA–PEEA). Force–displacement curves (a) using glass-co-polymer-PTFE and (b) PTFE-co-polymer-PTFE substrates respectively.

The adhesive strength of poly(PDMA–PEEA) polymers were determined using force–displacement curves obtained after lubricating adhesive polymer on to rectangular glass and PTFE sheet or PTFE and PTFE substrates pulled apart at an elongation speed of 1.0 mm min^−1^ at room temperature. The results (Fig. 3a and b) shows that on poly(PDMA–PEEA) coated PTFE – PTFE sheets or PTFE-glass substrates, the force increases initially quite steeply, and in that range, high force is required to overcome the friction and adhesion at the interface, as well as the internal stiffness of the adhesive polymer. Therefore, in the initial range (up to 5 mm), the PTFE sheet moves very slightly with respect to PTFE sheet or glass substrates. The maximum stress (MPa) decreases gradually with the increase of EEA content because of the increased viscosity of adhesive copolymer. The maximum stress (MPa) and displacement values are summarized in Table 3.

**Table 3:** Adhesive strength of poly(PDMA–PEEA) with different EEA contents coated on glass substrate and PTFE sheet (in the table, the values in parenthesis are for polymer coated on PTFE and PTFE substrates)

Sample	Max. stress [MPa]	Displacement [mm]
1 : 2.5	5.1 × 10^−2^ (3.9 × 10^−2^)	20.0 (22.4)
1 : 5	3.5 × 10^−2^ (2.6 × 10^−2^)	22.3 (24.0)
1 : 7.2	3.2 × 10^−2^ (2.5 × 10^−2^)	22.3 (26.3)
1 : 10	2.3 × 10^−2^ (2.4 × 10^−2^)	25.2 (41.4)

The differences in surface properties were also demonstrated by the changes in water contact angles measurements. The water contact angle of smooth PTFE sheets was 114°, while it was 70° after coating with adhesive copolymer, poly(PDMA–PEEA). The results of time dependence contact angle measurements shows the contact angle decreased from 70° to 30°. This suggests that the surface of PTFE became hydrophilic after coating with poly(PDMA–PEEA) which has catechol moieties. The effects of different weight ratios of monomers in poly(PDMA–PEEA) on surface properties of PTFE and time-dependence change in contact angles on glass substrates coated with adhesive polymers were also studied. The values are summarized in Table S1.† The measurement shows increase of hydrophilicity (decrease of contact angles) of adhesive polymer owing to the swelling of polymers.

**Fig. 4:**
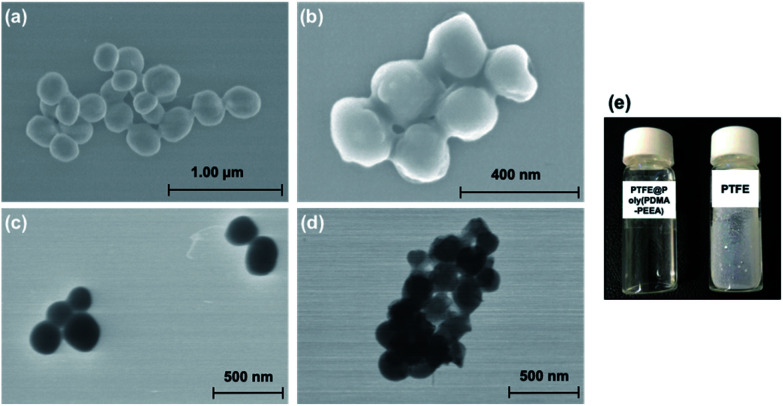
SEM images of (a) PTFE and (b) PTFE@poly(PDMA–PEEA); STEM images of (c) PTFE and (d) PTFE@poly(PDMA–PEEA); (e) shows the photograph of poly(PDMA–PEEA) coated PTFE particles and pristine PTFE particles in aqueous solution.

Scanning electron microscopy (SEM) and scanning transmission electron microscopy (STEM) images of the PTFE powdered samples and coated PTFE particles are shown in Fig. 4. In order to disperse PTFE particles in aqueous medium, the adhesive polymer was coated onto PTFE particles in an organic solvent. The uniform coating was ensured in SEM and STEM images where the original spherical morphologies of PTFE with diameter *ca.* 100–150 nm was clearly distinguishable from the crumpled surface of coated PTFE with diameter *ca.* 150–200 nm. Fig. 4c shows the photograph of poly(PDMA–PEEA) coated PTFE particles and pristine PTFE particles in aqueous medium.

**Fig. 5:**
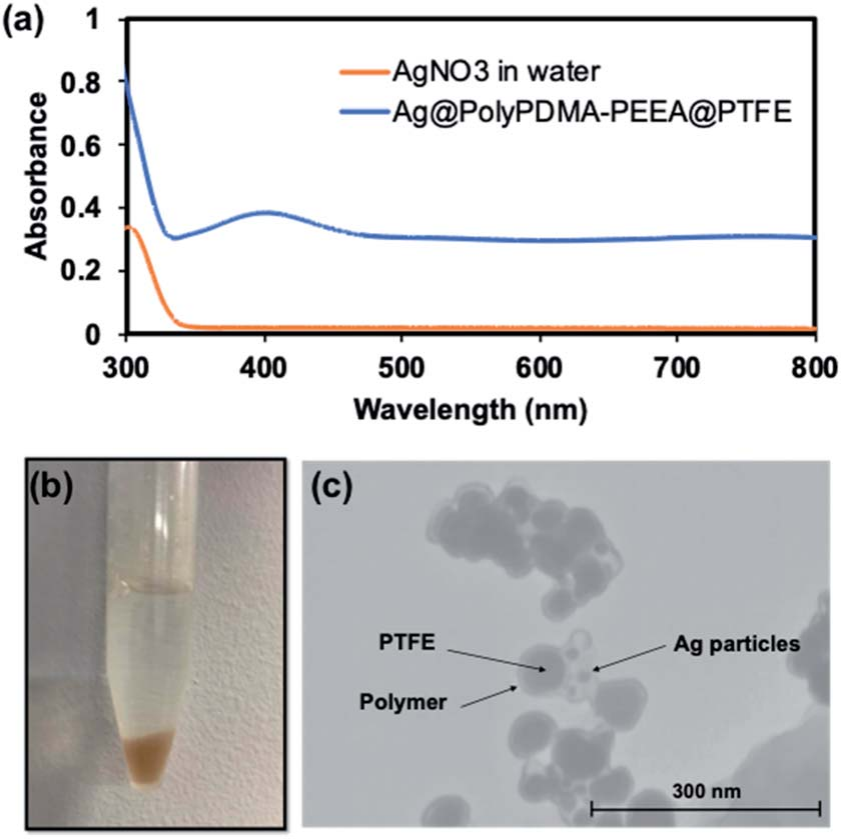
(a) shows UV-Vis spectra of THF solutions of poly(PDMA–PEEA) and PTFE@poly(PDMA–PEEA)@Ag nanoparticles, whereas, (b) photograph and (c) TEM images of PTFE@poly(PDMA–PEEA)@Ag nanoparticles dispersed in water.

Catechol moieties in adhesive polymer also acts as a reducing agent^33–35^ and can stabilize metal nanoparticles surfaces because of their strong adhesion properties. In this context, the reduction of silver nanoparticles was carried out using polymer-coated PTFE particles in the procedure followed. In brief, 2 mL of as-synthesized PTFE@poly(PDMA–PEEA) particles in distilled water (5 mg mL^−1^) was mixed with 2 mL of aqueous AgNO_3_ solution. The color of PTFE@poly(PDMA–PEEA) particles changed to orange within few minutes. The solution was kept at 25 °C for overnight in order to react catechol moieties from poly(PDMA–PEEA) completely. The mixture was subjected to centrifugation and washed with distilled water 3 times in order to remove excess of Ag ions. The particles were redispersed in water for further analysis. Fig. 5 shows the photograph of orange Ag nanoparticles adsorbed on the surface of PTFE@poly(PDMA–PEEA) suspended in water, and an absorption spectra of solution of PTFE@poly(PDMA–PEEA)@Ag and poly(PDMA–PEEA). As shown in Fig. 5b, the absorption peak at around 300 nm in both the spectra is attributed to absorption of catechol moieties in poly(PDMA–PEEA). Additionally, there is a strong absorption peak at around 400 nm in the spectrum of PTFE@poly(PDMA–PEEA)@Ag, which was attributed to the surface plasmonic resonance (SPR) due to reduction of Ag ions to Ag NPs. Fig. 5c shows TEM image of Ag NPs on the surface of poly(PDMA–PEEA) coated PTFE particles. Furthermore, the spectroscopic evidence (Fig. S5†) shows the uniform coating of PTFE particles by poly(PDMA–PEEA). This composite PTFE@poly(PDMA–PEEA) particles act as a nanoreactors for carrying out reduction of inorganic Ag ions without use of any additional reducing agents or surfactants as stabilizers as excess of catechol moieties in adhesive polymer that were not oxidized in reducing Ag ions adhered to the surface of Ag NPs and stabilized them.

In conclusion, inspired by biological mussel-adhesion phenomenon, we present the simple, mild and facile chemical route for the synthesis of biomimetic adhesive polymer poly(PDMA–PEEA) from DMA and EEA as starting monomers in presence of thermal initiator AIBN. As dopamine-based polymers have adhesive and mild reductant abilities for metal salts due to the presence of abundant catechol and amine groups, therefore, neither special surface modification procedures of templates nor additional toxic or reducing agents are needed in this procedure. The synthesized adhesive polymers were directly used for coating hydrophobic polytetrafluoroethylene (PTFE) particles as template spheres in an organic solvent, and then dispersing coated PTFE particles in an aqueous medium. Silver precursor ions Ag^+^ are added and absorbed onto the surfaces of poly(PDMA–PEEA)@PTFE composite spheres by the active catechol and amine groups of poly(PDMA–PEEA) coating. Meanwhile, the absorbed Ag^+^ ions are *in situ* reduced to Ag NPs and are home positioned. In other words, the adhesive polymer-coated PTFE composite particles were applied as a nanoreactor for generating silver metal nanoparticles (Ag NPs) without additional reducing agents as the catechol moieties in the copolymer acted as reductants and stabilizers for dispersing inorganic Ag NPs. It is possible to envisage that the composite nanostructure based on adhesive coating will contribute in simpler and facile models for surface modification of hydrophobic surfaces, and for inorganic NPs reductions eliminating the use of complex systems for catalytic reductions or miscellaneous applications.

## Conflicts of interest

There are no conflicts to declare.

## Supplementary Material

RA-010-C9RA10606E-s001
