# Supplementary material for: Biomimetic catechol-based adhesive polymers for dispersion of polytetrafluoroethylene (PTFE) nanoparticles in an aqueous medium
Source: RSC Adv. 2020 Jan 23;10(7):4058–63. doi: 10.1039/c9ra10606e (PMC9048857; doi:10.1039/c9ra10606e)
Supplement: RA-010-C9RA10606E-s001 [file RA-010-C9RA10606E-s001.pdf]

Electronic Supplementary Material (ESI) for RSC Advances.
This journal is © The Royal Society of Chemistry 2020

Biomimetic Catechol based Adhesive Polymers for Dispersion of Polytetrafluoroethylene (PTFE) Nanoparticles in an Aqueous Medium

*Manjit Singh Grewal, Hiroshi Yabu**

WPI-Advanced Institute of Materials Research (WPI-AIMR), Tohoku University, 2-1-1, Katahira, Aoba-Ku, Sendai 980-8577, Japan

Supplementary

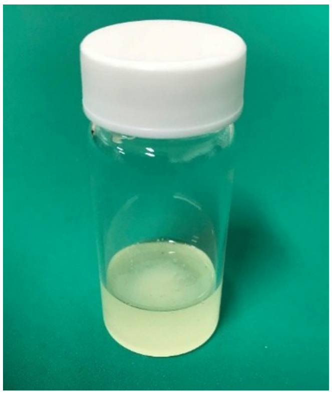

Figure S1. Representative photograph of adhesive polymer, poly(PDMA-PEEA) 1:10.

a.

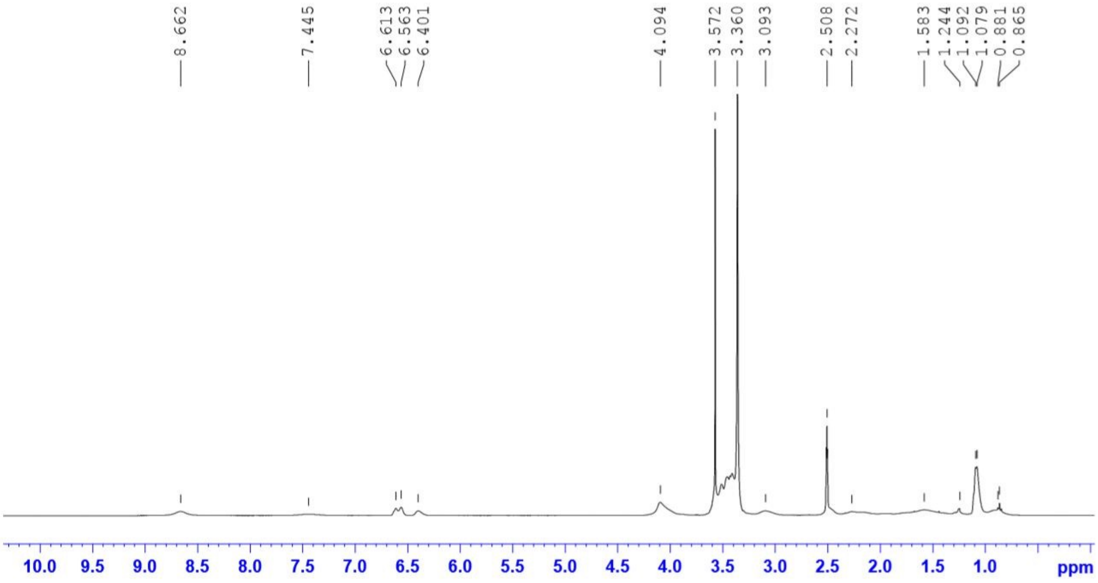

b.

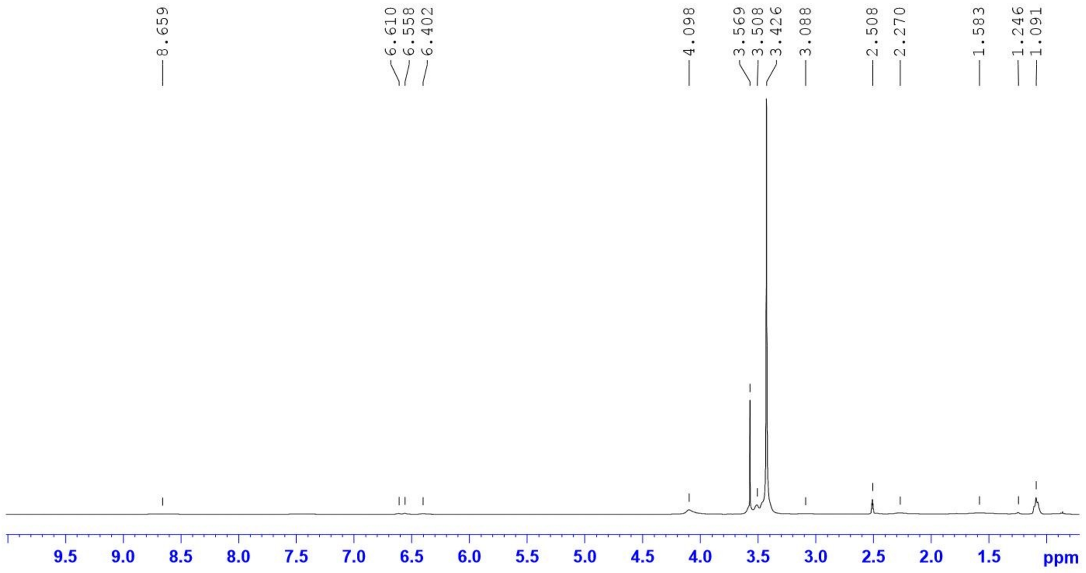

8.659
6.610
6.558
6.402
4.098
3.569
3.508
3.426
3.088
2.508
2.270
1.583
1.246
1.091
9.5 9.0 8.5 8.0 7.5 7.0 6.5 6.0 5.5 5.0 4.5 4.0 3.5 3.0 2.5 2.0 1.5 ppm

c.

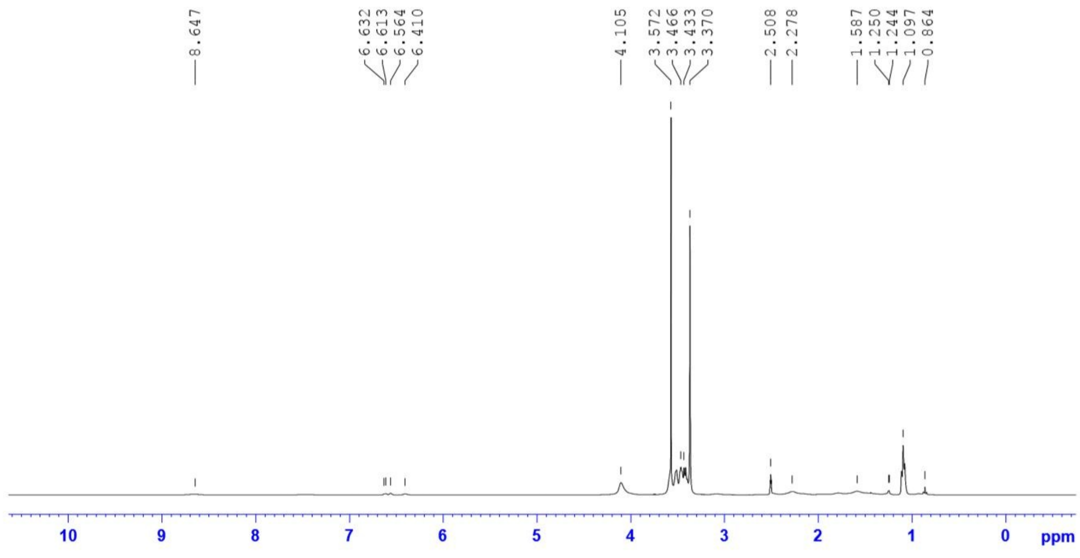

8.647
6.632
6.613
6.564
6.410
4.105
3.572
3.466
3.433
3.370
2.508
2.278
1.587
1.250
1.244
1.097
0.864
10 9 8 7 6 5 4 3 2 1 0 ppm

d.

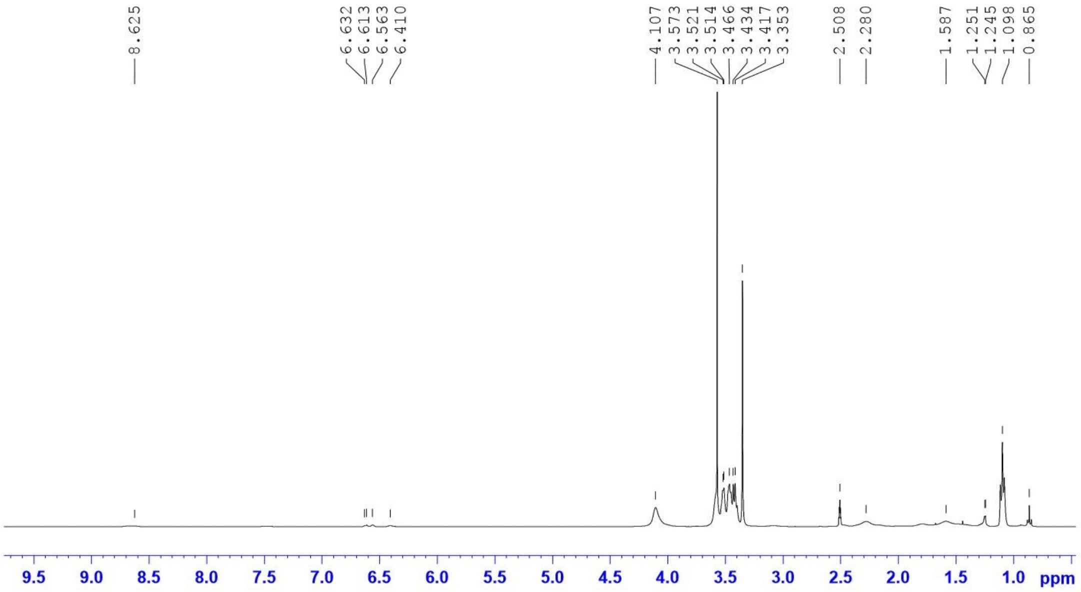

Figure S2: ^{1}H-NMR of adhesive polymer, poly(PMDA-PEEA) with DMA: EEA as (a) 1:2.5 (b) 1:5 (c) 1:7.2 (d) 1:10.

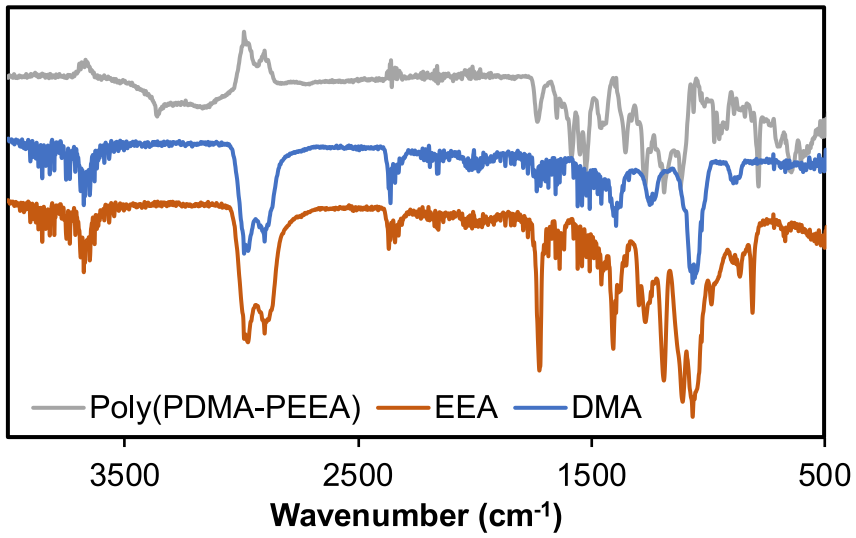

Figure S3: FT-IR spectra of EEA, DMA and copolymer poly(PDMA-PEEA).

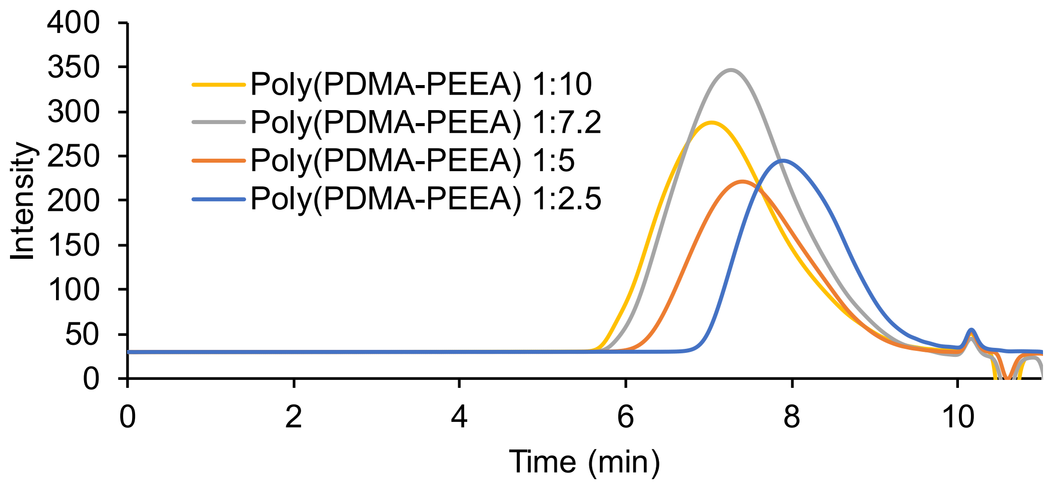

Figure S4: GPC results of adhesive polymer, poly(PMDA-PEEA).

Table S1: Time dependence contact-angle measurements of poly(PDMA-PEEA) with different EEA contents on glass substrates.

**Time (in minutes)**	**Polymer (1:2.5)** **CA±SD**	**Polymer (1:5.0)** **CA±SD**	**Polymer (1:7.2)** **CA±SD**	**Polymer (1:10)** **CA±SD**
**0**	77.2 ± 3.7	70.6 ± 7.3	72.7 ± 5.0	69.7 ± 5.2
**20**	75.7 ± 8.3	57.8 ± 6.7	62.6 ± 4.9	57.1 ± 8.5
**40**	69.4 ± 8.4	50.9 ± 17.5	58.2 ± 9.8	39.8 ± 14.6
**60**	53 ± 11.3	40.7 ± 9.0	37.9 ± 11.4	24.5 ± 3.0
**80**	52.5 ± 9.6	34.6 ± 5.3	32.6 ± 12.0	23.7 ± 4.3
**100**	45.3 ± 11.4	28.9 ± 11.8	28 ± 10.0	18.4 ± 1.9

**120**	34.7 ± 3.9	23.9 ± 5.1	21.4 ± 9.0	14.2 ± 2.1

a.

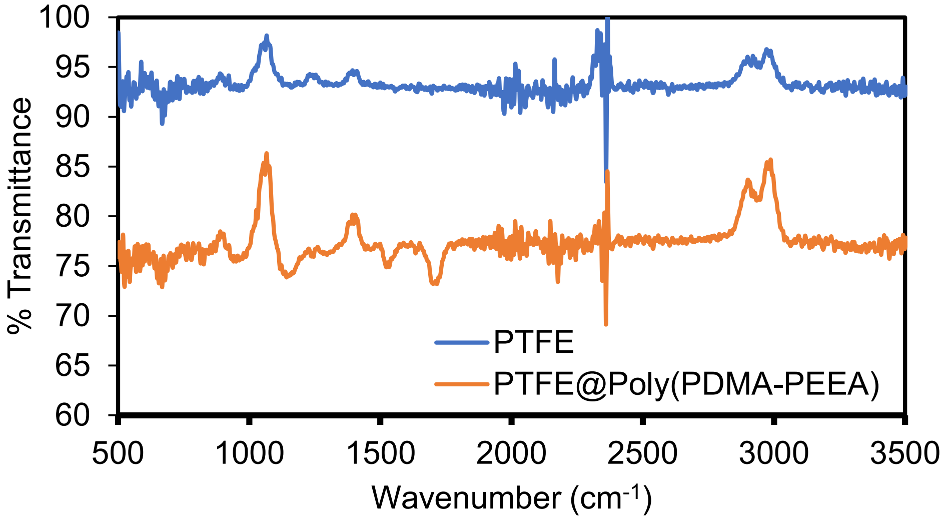

b.

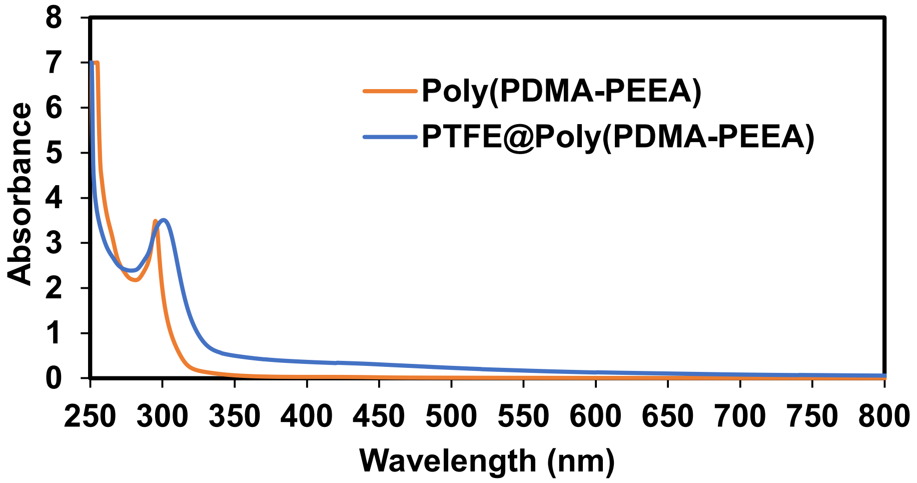

Figure S5 (a). FT-IR spectra of pristine PTFE and poly(PDMA-PEEA) coated PTFE. **(b)** UV-Vis spectra of poly(PDMA-PEEA) and poly(PDMA-PEEA) coated PTFE in THF.

The FT-IR spectra of pristine PTFE show peaks for the CF_3 (1250 cm^{-1}) and CF_2 (1190 cm^{-1}) groups, whereas in poly(PDMA-PEEA) coated PTFE the appearance of C=O (1715 cm^{-1}) strongly indicates the coating of PTFE by polymer. In addition, UV-Vis

spectra of poly(PDMA-PEEA) and poly(PDMA-PEEA)@PTFE shows the absorption peaks around 300 nm, which is attributed to the presence of catechol moieties.
